# Development of the multidimensional health perceptions questionnaire in English and Spanish

**DOI:** 10.1186/s41687-022-00512-4

**Published:** 2022-09-24

**Authors:** Shannon B. Juengst, Marlene Vega, Alexandra B. Holland, Susan Herrera, Robin T. Higashi, Maria Boix Braga, Alka Khera, Chung Lin Kew, Valeria Silva

**Affiliations:** 1 Brain Injury Research Center, TIRR Memorial Hermann, 1333 Moursund St., Houston, TX 77030 USA; 2 Department of Physical Medicine and Rehabilitation, University of Texas Southwestern Medical Center, Dallas, TX USA; 3 Department of Psychiatry, University of Texas Southwestern Medical Center, Dallas, TX USA; 4 Department of Population and Data Sciences, University of Texas Southwestern Medical Center, Dallas, TX USA; 5 Harold C. Simmons Comprehensive Cancer Center, Dallas, TX USA; 6 Department of Neurology, University of Texas Southwestern Medical Center, Dallas, TX USA; 7 School of Public Health, Texas A&M University, College Station, TX USA; 8 Perot Foundation Neuroscience Translation Research Center, Peter O’Donnell Jr. Brain Institute, University of Texas Southwestern Medical Center, Dallas, TX USA

**Keywords:** Health belief model, Psychometrics, Health literacy, Locus of control, Biopsychosocial

## Abstract

**Purpose:**

To develop the novel multidimensional health perceptions questionnaire (MHPQ), a self-reported assessment of health perceptions inclusive of (1) individuals beliefs about the causes and consequences of health conditions, benefits and barriers to maintaining and improving health, ability to accomplish health-related goals and control health circumstances, and the role of God and/or spirituality in health and healthcare, (2) anticipated discrimination in the healthcare systems, and (3) trust in healthcare providers and medicine, illustrated in our newly proposed Multidimensional Health Perceptions Conceptual Model.

**Methods:**

We developed an initial MHPQ_β_ item set, corresponding to domains of our conceptual model, using a patient-centered outcomes development approach. This include literature review, expert and end-user feedback, translation and language validation (specifically to Latin American Spanish), and cognitive interviewing.

**Results:**

The initial 104 items of MHPQ_β_ had excellent content validity, with a Content Validity Index of 98.1%. After expert (n = 13) feedback, translation and language validation, and cognitive interviewing among community-dwelling English-speakers (n = 5) and Spanish-speakers (n = 4), the final MHPQ_β_ comprised 93 items rated on a five-point agreement scale (1 = Strongly disagree to 5 = Strongly agree), with a reading grade level of 6th grade in English and 8th grade in Spanish.

**Conclusion:**

The MHPQ_β_ is a promising tool to assess individuals’ health perceptions. It has excellent content validity and good reading accessibility. Future work will establish the factor structure and final item set of the MHPQ.

## Introduction

Culturally competent, patient-centered, and family-centered practice requires that clinicians engage in open dialogue with the individuals they treat and their family members, accounting for their personal beliefs and preferences, cultural values, resources, and abilities when making treatment decisions and personalizing communication approaches and recommendations accordingly [1–3]. Newer conceptualizations of patient centeredness extend beyond just these immediate interpersonal interactions to include an understanding of how the healthcare system, as a whole, might treat individuals [2]. In parallel, culturally competent care has risen in prominence in direct response to systemically-driven racial and ethnic healthcare disparities and now also emphasizes prejudice, stereotyping, and social determinants of health [2]. Brief and valid measures that could provide information as a basis for such conversations could improve patient-provider communication within the bounds of time-restricted practice.

Early models of culturally competent care generally included the following components: (1) respecting that individuals’ health beliefs are legitimate and accepting that they affect health and healthcare efficacy; (2) taking a biopsychosocial (rather than biomedical) approach to healthcare, considering the individual as a whole rather than just the disease or condition being treated; (3) actively eliciting from individuals their beliefs about their illness or condition and its causes; and (4) communicating in clear and accessible language (both related to language fluency and health literacy level) [2]. This includes detailed understanding of real-world conditions that exert causal effects on health outcomes and treatment adherence, uptake, and efficacy, including personal beliefs about health and healthcare [4–6]. This places increasing demands on healthcare providers to understand patients within complex systemic contexts. Therefore, healthcare providers need efficient and effective tools to capture and understand individual health beliefs to practice patient-centered, family-centered, and culturally competent care. Further, our increasingly aging population and the shift in healthcare focus towards future self-management of chronic health conditions require that we also shift how we think about and account for the effect of health beliefs and abilities on health outcomes.

Despite recognition of the importance of health perceptions and how complex health perceptions are, existing measures narrowly evaluate health beliefs (e.g., focus only on locus of control) and are often limited in scope to specific conditions (e.g., stroke [7]). The World Health Organization’s Quality of Life Measure (the WHOQOL) includes “Spirituality /Religion/Personal Beliefs” as a measured domain, but it measures how a person’s personal beliefs (not necessarily related to health) give them strength to face difficulties or give meaning to their lives, rather than assessing what their health-related perceptions actually are [8]. Integrating multidimensional constructs to evaluate health perceptions in a single measure could allow clinicians to identify perceptions salient to specific individuals to improve intervention design, evaluation, and efficacy and to promote patient-centered and culturally competent care. Further, measures are rarely adapted for cultural and linguistic minorities, contributing to existing health disparities. Hispanic individuals are already underrepresented in health care research, despite already representing a large portion of the U.S. population and projections that they will make up 28% of the total U.S. population by 2060 [9]. Therefore, measures must be adapted for Spanish-speakers in the U.S. to provide valid and meaningful assessment that is the backbone of healthcare research and evidence-based care.

**Fig. 1:**
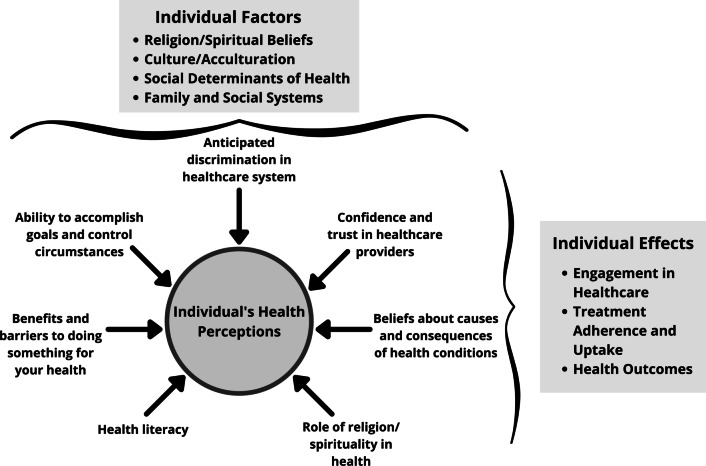
Multidimensional health perceptions model

To address the need for a person-centered, culturally sensitive, bilingual assessment of individual health perceptions, our objective was to develop a new health perceptions measure for both English- and Spanish-speakers that incorporates multiple health perceptions domains into a single measure. As a conceptual basis to guide item development for this new measure, we first developed the Multidimensional Health Perceptions Model (Fig. 1) by integrating established theories and drawing on concepts from existing measures.

### Multidimensional health perceptions model

We developed the Multidimensional Health Perceptions Model (Fig. 1) by integrating constructs from (1) the Health Belief Model [10] to explain factors involved in health behavior change, (2) Social Cognitive Theory [11] to explain individual factors that contribute to motivation and ability to change health behaviors, and (3) Ecological Systems Theory [12] to explain how social systems affect health behavior. While the Health Belief Model has provided a useful framework for understanding cognitive determinants of many health behaviors [13], it does not account for individual or environmental factors that facilitate or inhibit acceptance of health behaviors and impetus for change, such as trust in healthcare systems and providers. It also does not explain mediating factors, such as self-efficacy or health literacy, or provide strategies for health behavior change. Its predictive capability for health behavior change has also been challenged [13, 14].

A social cognitive approach to health promotion emphasizes effective self-management. Management of chronic health conditions often requires lifestyle or other behavioral changes. However, cues to action alone—such as a clinician providing health recommendations or education—do not necessarily result in behavior change. Health behavior change interventions must include self-management skills needed to translate recommendations or education into personalized and meaningful behavior change. These interventions also need to account for how cues to action are perceived by the individuals receiving them. Healthcare providers routinely give recommendations (i.e., cues to action) with the assumption that they can and will be followed. When they are not, providers often attribute lack of treatment adherence to patient attributes (e.g., lack of knowledge, intentional noncompliance)[15] rather than to the recommendations themselves or to the mode of delivery (e.g., instructions written at a high level of education). Even more disconcerting, negative descriptors and judgments are made more often in the electronic medical record notes of Black patients (than White patients) and women [16, 17]. Consistent with newer conceptualizations of patient-centered and culturally competent care, healthcare recommendations and patient-provider communication need to move beyond the Health Belief Model to account for biopsychosocial factors, including health literacy and systemic racism, that contribute to health and health behavior change.

An individual’s health perceptions reflect the complex interplay between numerous personal, psychosocial, and systemic factors that affect engagement in healthcare, adherence to and uptake of treatment and health behavior recommendations, and, ultimately, their health outcomes. Our model incorporates religious and spiritual beliefs, locus of control, and a multitude of social determinants of health that contribute to an individual’s health perceptions. We operationalize health perceptions as an individual’s beliefs about the causes and consequences of health conditions (Health Beliefs Model), the benefits and barriers to maintaining and improving health (Health Beliefs Model), their ability to accomplish health-related goals and control health circumstances (including locus of control and health literacy; Social Cognitive Theory), beliefs about the role of God and/or spirituality in health and healthcare (Ecological Systems Theory), anticipated discrimination they might experience in the healthcare system (Ecological Systems Theory), and trust in healthcare providers and medicine (Ecological Systems Theory). These latter factors are particularly important in light of clear evidence of systemic barriers to healthcare equity [18–22]. Understanding how social experiences might lead to mistrust of the healthcare system is critical for adapting how we communicate health information to populations that experience discrimination and inequity. For example, in the Coronavirus Tracking Study, individuals reported feeling that healthcare providers and staff judged them or discriminated against them, with Black adults, women, and individuals with low-income reporting this most often [23]. As another example, Latinx immigrants have reported experiencing chronic stress and anxiety from an anti-immigrant social environment characterized by anti-immigrant rhetoric, legal policies, and discrimination, resulting in distrust in community resources, poor mental health, and healthcare barriers [24]. However, “culture” does not equate with racial or ethnic group, as individuals within a broader racial or ethnic group represent a diversity of cultures and health-related needs and beliefs [25]. Continuing to attribute differences in outcomes to race or ethnicity, rather than directly measuring the underlying factors that we assume race and ethnicity are proxy measures [26] for (such as differences in culturally-related health beliefs or experience of microaggressions in the healthcare system), will only hinder culturally competent practice.

To develop items for a new measure that effectively and sensitively captures health perceptions across the domains outlined in our conceptual model, we took a multistep, patient-centered approach that relied on reviewing content from existing, theoretically-grounded measures and engaging relevant stakeholders—including content experts and people with relevant lived experiences—to provide feedback throughout the process. Herein, we present this process and results of the development of the initial item set for the Multidimensional Health Perceptions Questionnaire (MHPQ).

## Methods

### ***Development of the MHPQ***_***β***_

We developed the initial item set of the MHPQ in English and Spanish through an iterative process of literature review, end-user and expert feedback, and revision. We used patient-centered techniques (e.g., cognitive interviews) that our team has previously employed, ensuring relevance, cultural and conceptual equivalence, and accessibility for low literacy users [27]. This process included the following steps: (1) An initial item set was written to cover all components of our conceptual model; (2) An expert panel evaluated the relevance of these items to the conceptual model to establish the content validity of the MHPQ; (3) Items were translated to Spanish and underwent a rigorous language validation process to ensure conceptual and cultural equivalence; (4) We solicited direct feedback about specific items and health perceptions more broadly from primary English- and Spanish-speakers through cognitive interviews, presenting their qualitative perspectives to ensure we developed a person-centered and culturally sensitive measure; (5) We revised the item set in English and Spanish in response to steps 2–4. All participants in cognitive interviews provided verbal informed consent, per approval from the [REDACTED] Institutional Review Board.

#### Step 1: Creating initial item set

Initial proposed items for the MHPQ were generated by coauthors based on our conceptual model (see Fig. 1) and content review of other validated assessments, including the Health Belief Model [10], Everyday Discrimination Scale [28–30], Multidimensional Health Locus of Control Scales [31, 32], Irrational Health Belief Scale[33], All Aspects of Health Literacy Scale [34], God Health Locus of Control Scale [35], and Working Alliance Inventory [36]. We wrote items following these guidelines: (1) short sentences communicating a single concept; (2) language no higher than an 8th grade reading level; and (3) no double-barreled questions. In several cases, we wrote the same item two or three different ways, then determined which was better (in both English and Spanish) from end-users during cognitive interviews.

#### Step 2: Expert panel and content validity

The expert panel included n = 13 healthcare professionals, including social workers, clinical psychologists, and experts in public health and behavioral sciences. Experts ranged in age from 25 to 50 and in experience from 1 to 20 years. They represented Hispanic and non-Hispanic men and women across multiple racial groups. Several were bilingual in English and Spanish. Expert panel members rated the relevance of individual items on a 5-point ordinal scale (1 = Not at all relevant to 5 = Highly Relevant) and provided qualitative feedback regarding content and wording. We calculated average relevance scores per item. A final content validity index was calculated as the number of items with average relevance scores > 3.5 divided by the total number of items, with a threshold of 80% set as an indicator of good content validity [37, 38].

#### Step 3: Translation and language validation

Translation and language validation of initial and new items into Spanish was conducted by a committee of diverse native speakers from different countries of origin who looked for accuracy and conceptual equivalence in the translation. Items were translated and back translated by native bilingual speakers on our team, who also represent different countries of origin (United States, Mexico, Puerto Rico, and Venezuela).

#### Step 4: Cognitive interviews

Participants were recruited via a research registry and through referrals and were compensated for their participation. Cognitive interviews (1 h duration) with both English-speaking (n = 4) and Spanish-speaking (n = 5) community-member participants probed wording, meaning, and content of several survey items to assess clarity of language and concepts. The purpose of cognitive interviews is to ensure that the meaning of survey language as understood by participants matches the meaning intended by investigators. Semi-structured interview questions then further probed participants’ health beliefs and locus of control. For example, they assessed whether participants prioritized certain factors that influence health and illness over others in certain situations, and how participants felt about being in control of their health.

#### Step 5: Revision of items in English and Spanish

Our team revised items based on expert and consumer feedback and the language validation process. New items were developed based on expert recommendations and feedback from participants during cognitive interviews. To develop new items, we consulted other belief and perception scales. We selected which version of an item was best based on cognitive interviews. All new and revised items underwent a similar language validation process as described above. We evaluated the required reading level in both languages using the modified SMOG Index [39, 40], with a target of ≤ 8th grade reading level. We found that the initial Spanish items indicated an average 11th grade reading level, so our team reviewed sentence length and number of polysyllabic words to reduce the literacy level to ≤ 8th grade in Spanish, while maintaining conceptual equivalence.

## Results

### Development and content validity

The expert panel reviewed and rated 104 initial items on the MHPQ_β_, which demonstrated an excellent Content Validity Index of 98.1% [37] and high average relevance of 4.2 (out of 5). Cognitive interviews with n = 9 community-member participants (see Table 1 for demographics) informed selection of best wording for several items and development of new items. During the cognitive interviews and via expert panel feedback, participants and experts suggested additional concepts that were relevant and for which new items were developed. These included the effects of a positive attitude, the effects/attitudes of others, the role of miracles in health, the effects of the surrounding environment on individual health, the role of stigma regarding certain diseases/conditions, being seen as a burden by healthcare providers, the use of home remedies, the effect of having a healthcare provider that is similar to the individual, and compensatory beliefs. We generated new questions based on expert panel feedback and content review of other validated assessments, reiterating steps of our initial item development previously described. Other validated assessments reviewed were the Powe Fatalism Inventory [41], the revised life orientation test (LOT-R) [42], the Multidimensional Health Locus of Control Scales [31, 32], the Compensatory Health Beliefs Scale [43], the Supernatural Belief Scale (SBS) [44], the General Health Questionnaire [45], the Wagnild and Young Resilience Scale [46], and instruments assessing the patient-doctor relationship [47].

**Table 1:** Demographics for cognitive interviews

	English (n = 4)	Spanish (n = 5)
Gender (Women)	25%	80%
Age range	34–91	26–69
Education range	13–16	6–13
Race (White)	75%	100%
Ethnicity (Hispanic)	25%	100%

The final MHPQ_β_ included 91 items. Twenty-one of these items were modified from the original wording based on expert panel or cognitive interview feedback. Fourteen of these items were new based on feedback from cognitive interview participants. Twenty-seven items were removed based on expert panel ratings of relevance and from collapsing multiple versions of the same item after probing during cognitive interviews. The final item set and instructions had a modified SMOG reading level of 6th grade in English and 8th grade in Spanish.

### Qualitative perspectives from consumers

Three themes emerged from the qualitative inquiry about health beliefs and locus of control. First, participants cited multiple factors that influence health and illness, including self-behaviors, God/spirituality, genetics, and family/social support. Of these, many felt that a plurality of factors affected health in general. Second, participants felt that different factors played a role in different illnesses. For example, many felt that self-behavior was a primary factor for diabetes, but cited a variety of factors (e.g., God, genetics, one’s mental outlook, and support system) as being relevant in the case of cancer or depression. Third, participants disagreed about whether having control over one’s health was empowering or burdensome. For example, some participants stated that knowing that self-behaviors were a factor in their health felt empowering; others said they felt guilty and isolated by their health situation if they felt they hadn’t done an adequate job in taking care of their health.

## Discussion

We developed the novel Multidimensional Health Perceptions Questionnaire (MHPQ), a self-report measure of health perceptions that integrates beliefs about individual, psychosocial, and systemic factors that affect healthcare engagement, treatment adherence and uptake, and outcomes. We developed the Multidimensional Health Perceptions Model as a conceptual framework to guide content development. We then developed items for the MHPQ to capture individual health perceptions, employing patient-reported outcomes development techniques to ensure relevance, accessibility, and scientific validity. Our approach was consistent bilingual participatory research processes deemed critical for moving towards equity for Hispanic/Latinx persons in the U.S [48].

The MHPQ demonstrated excellent content validity, with experts indicating that its items were highly relevant to and appeared to measure the concepts that they were intended to measure, as illustrated in the Multidimensional Health Perceptions Model. Beyond the items initially developed to capture constructs in our conceptual model, end-users and experts recommended two other constructs relevant to health perceptions that were not captured by our model, namely the effects of others or attitudes of others on health (e.g., the “evil eye”) and the role of stigma surrounding certain diseases/conditions (e.g., embarrassment about one’s condition). We will determine whether and how these themes map to the original conceptual model after pilot testing and conducting factor analysis on the MHPQ_β_. The initial MHPQ_β_, including instructions for completing the measure, has a 6^th^ and 8^th^ grade reading level in English and Spanish respectively. By including end-users in the item-development process and employing robust methods to ensure accurate, conceptually equivalent, and accessible (i.e., low reading level) English and Spanish language versions of the MHPQ, we have built the foundation necessary for a valid, reliable, and clinically useful measure of individual health perceptions.

To achieve the intended purpose of the MHPQ, further study will next identify its factor structure and determine the extent to which it captures the domains of the Multidimensional Health Perceptions Model. Through factor analysis, we can identify subscales to comprehensively characterize individuals’ most salient health perceptions. Once unidimensional subscales are established, further psychometric analysis, such as Rasch Analysis and Differential Item Functioning, could be conducted to evaluate scale properties and potential item-level biases based on personal factors such as ethnicity, gender, or age. Subscale scores could be used to establish unique health perceptions profiles (i.e., patterns of health perceptions) that could inform multilevel interventions, including interventions for individuals, care partners, and families, interventions for healthcare providers and care teams, and interventions directed at higher system levels (e.g., service delivery, policy) within specific institutions. Effectiveness of implementing the MHPQ in clinical practice to improve patient-provider communication and health outcomes would need to be tested in future pragmatic implementation trials.

### Limitations

While we employed a rigorous patient-centered outcomes approach, there are limitations to consider. Although our expert panel consisted of native English and Spanish speakers from diverse cultural backgrounds, the variability of the Spanish language across cultures and contexts makes it challenging to create a universal Spanish translation. Additionally, due to staff availability and time constraints, the expert panel consisted of English and Spanish speakers only, so we were unable to translate to other common languages in the U.S. like Chinese, French, Tagalog, Arabic, or numerous others [49]. Similar approaches to language translation and validation of the MHPQ are needed to ensure its cultural relevance to individuals who speak these other languages. After revision, the MHPQ indicates a SMOG reading level of 6th grade in English and 8th grade in Spanish. While the average American reads at the 8th grade level [50], this does not account for the variability of individual health literacy levels, and some individuals have lower literacy levels than average. Therefore, the MHPQ may still be at too high of a reading level for some individuals and therefore may not accurately capture their health perceptions. While we included persons with lived experience through cognitive interviews, and our expert panel included diverse individuals with regard to age, race, ethnicity, health conditions, and experience as informal care partners, we did not specifically have representation of ‘experts by experience’ in our expert panel. Additionally, although shortened from the initial 104 items, the current 91-item questionnaire may still be too extensive for use in some clinical situations. However, one purpose of the planned factor analysis is to reduce items so that the MHPQ has greater clinical utility. Further research is needed to assess the feasibility of using the MHPQ in “real-world” settings.

### Conclusions

We developed the Multidimensional Health Perceptions Questionnaire to measure the complex nature of health perceptions using a single measure. Included items capture the following components illustrated in the Multidimensional Health Perceptions Model: (a) expected discrimination in healthcare systems, (b) confidence and trust in healthcare providers and medicine, (c) beliefs about causes and consequences of health conditions, (d) role of religion/spirituality in health, (e) health literacy, (f) benefits and barriers to doing something for your health, and (g) ability to accomplish health-related goals and control circumstances. The MHPQ could be used to: (1) identify health perceptions salient to specific individuals, (2) improve treatment uptake, efficacy, and maintenance, and (3) promote patient-centered and culturally competent care.

## Data Availability

The datasets used and/or analysed during the current study are available from the corresponding author on reasonable request.
